# The association between graduated driver licensing laws and travel behaviors among adolescents: an analysis of US National Household Travel Surveys

**DOI:** 10.1186/s12889-016-3206-7

**Published:** 2016-07-27

**Authors:** Motao Zhu, Peter Cummings, Songzhu Zhao, Thomas Rice

**Affiliations:** 1 Center for Injury Research and Policy, The Research Institute at Nationwide Children’s Hospital, 700 Children’s Drive, Columbus, OH 43205 USA; 2 Injury Control Research Center, West Virginia University, Morgantown, WV USA; 3 School of Public Health and Harborview injury Prevention & Research Center, University of Washington, Seattle, WA USA; 4 Center for Biostatistics, The Ohio State University, Columbus, OH USA; 5 Safe Transportation Research & Education Center, University of California Berkeley, Berkeley, CA USA

**Keywords:** Legislation, Policy, Adolescent, Transportation

## Abstract

**Background:**

Young novice drivers have crash rates higher than any other age group. To address this problem, graduated driver licensing (GDL) laws have been implemented in the United States to require an extended learner permit phase, and create night time driving or passenger restrictions for adolescent drivers. GDL allows adolescents to gain experience driving under low-risk conditions with the aim of reducing crashes. The restricted driving might increase riding with parents or on buses, which might be safer, or walking or biking, which might be more dangerous. We examined whether GDL increases non-driver travels, and whether it reduces total travels combining drivers and non-drivers.

**Methods:**

We used data from the US National Household Travel Survey for the years 1995–1996, 2001–2002, and 2008–2009 to estimate the adjusted ratio for the number of trips and trip kilometers made by persons exposed to a GDL law, compared with those not exposed.

**Results:**

Adolescents aged 16 years had fewer trips and kilometers as drivers when exposed to a GDL law: ratio 0.84 (95 % confidence interval (CI) 0.71, 1.00) for trips; 0.79 (0.63, 0.98) for kilometers. For adolescents aged 17 years, the trip ratio was 0.94 (0.83, 1.07) and the kilometers ratio 0.80 (0.63, 1.03). There was little association between GDL laws and trips or kilometers traveled by other methods: ratio 1.03 for trips and 1.00 for kilometers for age 16 years, 0.94 for trips and 1.07 for kilometers for age 17.

**Conclusions:**

If these associations are causal, GDL laws reduced driving kilometers by about 20 % for 16 and 17 year olds, and reduced the number of driving trips by 16 % among 16 year olds. GDL laws showed little relationship with trips by other methods.

## Background

Traffic crashes are a major source of morbidity and mortality around the world, causing 20–50 million injuries and 1.2 million fatalities every year [1]. In the United States (US), traffic crashes accounted for 26 % (2,163/8,434) of the deaths of teenagers aged 16–19 years in 2013, and are the leading cause of death among teenagers [2]. Young novice drivers have crash rates higher than any other age group; per kilometers driven, the crash rate for 16-year-old drivers is approximately four times greater than that for drivers aged 30–59 years in the US [3]. To address this problem, graduated driver licensing (GDL) laws were first introduced in Florida in 1996, and all 50 states and the District of Columbia (DC) implemented some form of GDL by January 2012 [4, 5]. These laws typically have three phases [6]. During the extended learner permit phase, adolescents can only drive when supervised by a fully licensed driver. During the intermediate phase, nighttime driving and, in many states, the carrying of young passengers are both restricted. During the full licensure phase, unsupervised driving is permitted at all times.

Previous research has focused on crash reductions related to GDL implementation [6–11]. Few studies have tried to quantify the extent to which GDL laws affect driving, the use of alternative transportation (riding as a passenger, use of buses, bicycling, and walking), or the total number of trips [12, 13]. GDL laws allow adolescents to gain experience driving under low-risk conditions with the aim of reducing crashes. As GDL laws delay full licensure and restrict driving privileges, they might increase the use of alternative transportation to replace driving. Adolescents in California were reported to use the following transportation alternatives to adapt to the nighttime and passenger restrictions: have a parent or older adult as a supervising passenger; use of walking, biking, and bus; move their travel time to daytime; or violate the restriction [12]. Shifting to riding with parents or use of bus would be far safer, as would driving alone in the daytime by rearranging the time or event [14, 15]. However, shifting travel to walking or biking could be dangerous based on per trip fatality rates [16]. The California study was based on one state [12], and the association between GDL laws and alternative transportation was not quantified. Therefore, the objective of our study was to estimate, using nationally representative survey data, the association between GDL laws and both the number of trips and travel distances made by adolescents as passengers, bus riders, bicyclists, and pedestrians. We also examined the total travels combining drivers and non-drivers.

## Methods

### Data about annual trips and kilometers of travel

Estimates of annual trips and kilometers of travel were obtained from the 1995–1996, 2001–2002, and 2008–2009 National Household Travel Survey (NHTS) [17]. Respondents were a sample of non-institutionalized US civilians who were asked to keep a diary about all trips during a randomly assigned 24-h day, including transportation method. A trip was defined as a journey in which the respondent went from one address to another by any means [17]. If an adolescent drove from home to school, later drove from school to a grocery store, and then drove home, this would be counted as three trips. Each respondent was assigned a weight for their selection probability, adjusted for non-response and the presence of multiple household phones [17].

### Classifying graduated driver licensing

Presence of graduated driver licensing was determined for laws with a learner permit phase ≥ 3 months, plus an intermediate phase restriction on nighttime driving or number of young passengers [4]. Florida was the first U.S. state to implement GDL on July 1st, 1996. By 2002, 34 states and DC implemented GDL. By 2009, 45 states and DC implemented GDL. We classified NHTS respondents as exposed to a GDL law at the time of their trip diary if they were 16 or 17 years old and their state had a GDL law.

### Statistical analysis

At first, we estimated the average annual trips and kilometers for three age groups (16, 17 and 20–24 years old) and the three survey periods. As participants in the surveys are randomly selected, and their travel day is randomly selected throughout the season and day of the week, the estimates summarized from the trip diaries can represent the national average when incorporating sampling weights. We used the survey jackknife weights to compute the average trip counts and kilometers and the related variance.

We further estimated the average annual trips and kilometers according to GDL status, age groups and survey periods. To estimate the adjusted ratio of average annual number of trips or kilometers when exposed to GDL compared with not being exposed, we used variance weighted least squares linear regression [18, 19] with the log of average trips or kilometers in a year as the outcome and presence of a GDL law as the explanatory variable. The variance used, from the delta method [20], was the variance of the average annual number of trips divided by the average annual trips squared. The regression model included two indicator variables for the three driver age categories (16, 17, and 20–24 years old) and two indicator variables for the three survey periods. Drivers 20–24 years of age were included to help adjust for non-GDL factors that influence the number of trips over time, such as changes in the economy or traffic enforcement [13, 21, 22]. Relative to ages 16–17 years, the crash rate for ages 20–24 is much lower, principally due to increased psychological and physiocognitive maturation and increased driving experience [23–25]. In addition, this group was close to ages 16–17 years and probably not affected by graduated driver licensing in the US. Our regression used the trends among two adolescent ages and the trends among older drivers to jointly account for temporal trends in trips or kilometers independent of GDL laws. We used tests of interaction to evaluate whether any association between GDL laws and non-driver trip counts or kilometers varied by transport mode: car passenger, bus rider, bicyclist, and pedestrian. We initially included these potential confounders (unemployment rate, per capita income, and gasoline price) in the model, but none met the confounding criteria (changing the coefficient for drivers of age 16 by more than 10 %). Therefore, they were not included in the final model.

## Results

Trip diary data was available for 6,406 16-year-olds, 5,998 17-year-olds, and 16,471 persons age 20–24 years. The number of trips and trip kilometers as a driver and as a passenger in car or pick-up truck decreased over time for adolescents (Table 1). Bus trip counts changed little over time for those age 16 years but increased for those age 17 years. There were few bicycle trips. Walking trips increased over time.

**Table 1:** Annual trips: number and kilometers, by age and year^a^

Person type	Age (yr)	Year	Average number of annual trips (95 % CI)	Average annual kilometers (95 % CI)
Driver of passenger vehicle	16	1995-1996	521 (444 – 598)	4,588 (3,814 – 5,364)
		2001-2002	518 (450 – 586)	5,208 (4,297 – 6,117)
		2008-2009	321 (268 – 375)	2,729 (2,115 – 3,343)
	17	1995-1996	897 (802 – 993)	9,685 (7,326 – 12,046)
		2001-2002	771 (695 – 848)	9,065 (7,047 – 11,082)
		2008-2009	624 (560 – 688)	6,025 (5,189 – 6,862)
	20-24	1995-1996	1,152 (1,104 – 1,201)	17,263 (16,032 – 18,493)
		2001-2002	1,038 (1,001 – 1,075)	17,424 (16,245 – 18,602)
		2008-2009	943 (899 – 987)	14,841 (13,770 – 15,913)
Passenger in passenger vehicle	16	1995-1996	779 (692 – 867)	10,564 (7,747 – 13,378)
		2001-2002	720 (649 – 790)	8,913 (7,733 – 10,092)
		2008-2009	560 (509 – 611)	9,384 (7,678 – 11,090)
	17	1995-1996	715 (641 – 788)	10,372 (7,269 – 13,475)
		2001-2002	580 (507 – 652)	8,779 (7,131 – 10,429)
		2008-2009	357 (313 – 400)	7,792 (5,615 – 9,971)
	20-24	1995-1996	361 (328 – 394)	7,269 (6,090 – 8,449)
		2001-2002	332 (306 – 357)	6,869 (5,860 – 7,879)
		2008-2009	199 (177 – 220)	3,697 (3,045 – 4,350)
Bus rider	16	1995-1996	117 (93 – 141)	1,421 (995 – 1,846)
		2001-2002	122 (99 – 146)	1,798 (1,136 – 2,457)
		2008-2009	114 (95 – 134)	1,831 (1,308 – 2,354)
	17	1995-1996	63 (47 – 78)	1,054 (665 – 1,444)
		2001-2002	76 (57 – 95)	1,231 (911 – 1,551)
		2008-2009	130 (105 – 155)	2,412 (1,622 – 3,204)
	20-24	1995-1996	29 (22 – 35)	431 (275 – 586)
		2001-2002	32 (25 – 39)	475 (280 – 669)
		2008-2009	32 (23 – 41)	462 (277 – 647)
Bicyclist	16	1995-1996	19 (9 – 29)	55 (14 – 95)
		2001-2002	12 (5 – 18)	37 (3 – 71)
		2008-2009	13 (9 – 18)	31 (19 – 43)
	17	1995-1996	14 (5 – 23)	18 (5 – 32)
		2001-2002	14 (4 – 24)	47 (8 – 84)
		2008-2009	9 (5 – 12)	24 (10 – 37)
	20-24	1995-1996	16 (9 – 23)	61 (11 – 111)
		2001-2002	6 (4 – 9)	16 (8 – 24)
		2008-2009	14 (8 – 21)	92 (40 – 142)
Pedestrian	16	1995-1996	145 (108 – 181)	164 (97 – 232)
		2001-2002	157 (128 – 185)	161 (129 – 192)
		2008-2009	196 (155 – 236)	246 (156 – 336)
	17	1995-1996	108 (82 – 134)	92 (64 – 117)
		2001-2002	127 (91 – 162)	177 (116 – 237)
		2008-2009	150 (114 – 186)	164 (126 – 204)
	20-24	1995-1996	99 (85 – 113)	80 (68 – 95)
		2001-2002	137 (121 – 153)	145 (122 – 169)
		2008-2009	124 (107 – 141)	140 (114 – 164)

Abbreviation: *CI* confidence interval

^a^ Data from the National Household Travel Survey

Adolescents aged 16 years had fewer trips and kilometers as drivers when exposed to a GDL law: ratio 0.84 (95 % confidence interval (CI) 0.71, 1.00) for trips; 0.79 (0.63, 0.98) for kilometers (Table 2). Adolescents aged 17 years showed little change in the number of driver trips, but a reduction in driving kilometers when exposed to a GDL law: ratio 0.94 (0.83, 1.07) for trips; 0.80 (0.63, 1.03) for kilometers. There was little association between GDL laws and trips or kilometers by other methods: ratio 1.03 for trips and 1.00 for kilometers for age 16; 0.94 for trips and 1.07 for kilometers for age 17. The interaction tests did not suggest that the GDL associations with non-driver trips or kilometers varied between travel mode subgroups (car passenger, bus rider, bicycle, pedestrian) at age 16 (*p* = .94 for trips, .13 for kilometers) or age 17 (*p* = .17 for trips, .29 for kilometers). GDL laws were not associated with a reduction in all trips (driving and non-driving) for those age 16 (ratio: 0.98 [0.91, 1.05] and those age 17 (ratio: 0.95 [0.89, 1.01]). Overall trip kilometers might decrease about 10 % for both age groups, but the 95 % confidence intervals included one.

**Table 2:** Adjusted ratios comparing the annual trip counts and trip kilometers before and after graduated driver licensing^a^

	Adjusted ratio^b^ (95 % CI)			
	Trips		Kilometers	
	Age 16	Age 17	Age 16	Age 17
Driver	0.84 (0.71, 1.00)	0.94 (0.83, 1.07)	0.79 (0.63, 0.98)	0.80 (0.63, 1.03)
Other methods	1.03 (0.93, 1.14)	0.94 (0.84, 1.06)	1.00 (0.82, 1.22)	1.07 (0.84, 1.35)
Passenger in passenger vehicles	1.09 (0.96, 1.25)	0.90 (0.77, 1.04)	0.92 (0.73, 1.15)	1.00 (0.76, 1.31)
Bus rider	1.04 (0.81, 1.35)	1.26 (0.94, 1.67)	1.45 (0.99, 2.11)	1.24 (0.85, 1.80)
Bicyclist	1.16 (0.61, 2.19)	1.04 (0.48, 2.27)	0.75 (0.34, 1.66)	2.44 (0.97, 6.13)
Pedestrian	1.01 (0.78, 1.31)	1.14 (0.83, 1.55)	0.84 (0.61, 1.15)	1.09 (0.76, 1.55)
All trips	0.98 (0.91, 1.05)	0.95 (0.89, 1.01)	0.90 (0.78, 1.04)	0.91 (0.76, 1.09)

Abbreviation: *CI* confidence interval

^a^Data from the US National Household Travel Survey

^b^Adjusted ratios compare the average annual number of trips or trip kilometers by respondents exposed to graduated driver licensing with those not exposed, adjusted for changes over time. Exposure to graduated driver licensing means living in a state that had graduated driver licensing laws

## Discussion

Graduated driver licensing was associated with a 16 % reduction in driving trips and 21 % reduction of driving kilometers for age 16 years. Adolescents aged 17 years showed little reduction in driving trips, but a 20 % reduction in driving kilometers. These results suggest that adolescents drive less during the extended learner permit phase of GDL, perhaps because they have to drive under adult supervision and adult drivers, usually parents [25], are not always available. Adolescents also may drive less during the intermediate GDL phase, because of restrictions on night driving [12].

We found that GDL laws were not associated with a reduction in the frequency of trips by all driving and non-driving methods. These findings are compatible with a survey of California adolescents in which most reported that GDL restrictions had little impact on their activities [12]. Only 5 % of adolescents reported that the nighttime restriction prevented them from doing the things they wanted to do by a lot [12]. Approximately 91 % of licensed adolescents did not feel much inconvenience by the night and passenger restrictions in California [12].

We found little evidence in this data set that GDL laws influenced the frequency of trips by other modes. The California survey found the following ways adolescents adapted to the nighttime driving restriction: had parents or other adults as passengers (59 %), drove earlier (58 %), rearranged the event (45 %), drove at night despite the restriction (44 %), and walked or rode a bus or bicycle (31 %) [12]. The same survey found the following ways they adapted to the passenger restriction: drove alone (49 %), had parents or other adults as passengers (44 %), drove with passenger(s) despite the restriction (31 %), rearranged the event (21 %), and walked or rode a bus or bicycle (18 %) [12]. This showed that many trips restricted by graduated driver licensing were still carried out by adolescent drivers who moved the trip to daytime, drive alone, or had a parent or an older adult as a supervising passenger, thereby reducing the need to use alternative transportation to replace driving. Some adolescents simply did not comply with the GDL law and violated driving restrictions. A survey of North Carolina teenage drivers found that 23 % of teenagers violated the night restriction at least once, and 34 % of teenagers violated the passenger restriction [26]. An analysis of 2006–2009 U.S. nighttime driver fatalities among teenagers aged 15 – 17 years reported that 19 % of teenagers were non-compliant with the nighttime restriction [27].

From 2001–2002 to 2008–2009, the average number of annual trips and kilometers for drivers/passengers decreased for all three age groups (16, 17, 20–24). This might be linked with the economic recession and high unemployment during 2008–2009 [28]. By including persons aged 20–24 years in our model, and adjusting for temporal changes in crash rates over time among those covered and not covered by a GDL law in each teenage age group, we sought to control for non-GDL factors that influence travel over time [13, 21, 22, 28].

## Limitations

Our classification of GDL program is simply presence/absence, and we did not examine the strength of GDL programs or specific components such as minimum hours of practice, nighttime restriction, and passenger restriction. Although we had trip information from 12,404 adolescents, the confidence intervals around our estimates were wide due to the survey sampling design. Our ability to adjust for temporal trends in trip occurrence was limited by the small number of survey periods. The sampling scheme did not allow us to adjust for state or the availability of public transportation, which may introduce some bias in our estimates due to regional variations in trip frequency or kilometers of travel. Lastly, our research is an ecological study based on state, but not an individual-level analysis. Ecological bias may exist.

## Conclusion

GDL laws were associated with reduced trips and travel kilometers by adolescents as drivers. We found no evidence that GDL laws were associated with notable changes in the occurrence of trips as passengers, bus riders, bicyclists, or pedestrians. Future research is warranted to investigate the specific influence of GDL components such as nighttime restriction and passenger restriction.

## Abbreviations

CI, confidence interval; GDL, graduated driver licensing; NHTS, National Household Travel Survey; US, United States
